# Analyzing the Metabolomic Profile of Yellowtail (*Seriola quinquerdiata*) by Capillary Electrophoresis–Time of Flight Mass Spectrometry to Determine Geographical Origin

**DOI:** 10.3390/metabo11110793

**Published:** 2021-11-20

**Authors:** Jiyoung Shin, Junho Yang, Eunji Cha, Hyunsuk Kim, Yoonhyeung Lee, Soi Kim, Iseul Choi, Jiyoung Yang

**Affiliations:** 1 Institute of Food Science, Pukyong National University, Busan 48513, Korea; nadia83@naver.com; 2 Department of Food Science & Technology, Pukyong National University, Busan 48513, Korea; yjunho9@gmail.com (J.Y.); cnj429@naver.com (E.C.); zrepsriy@naver.com (H.K.); ps2leeyh@naver.com (Y.L.); soi117@naver.com (S.K.); ciseul@icloud.com (I.C.)

**Keywords:** yellowtail, metabolomics, geographical origin, discrimination method, CE-TOF/MS

## Abstract

Country-of-origin violations have occurred in which some merchants have fraudulently sold cheap Japanese yellowtail (*Seriola quinqueradiata*) by presenting them as domestic Korean products. There are many methods for determining the origins of marine organisms, such as molecular genetic methods and isotope analysis. However, this study aimed to develop a method for determining the origins of aquatic products using metabolite analysis technology. Ten yellowtail each from Korea and Japan were analyzed by capillary electrophoresis–time of flight/mass spectrometry (CETOF/MS). Hierarchical cluster analysis (HCA) and principal component analysis (PCA) results showed highly differing aspects between the Korean and Japanese samples. In the tricarboxylic acid (TCA) cycle, citric, malic, oxaloglutaric, and fumaric acids exhibited significant differences between Korean and Japanese yellowtail. Sixteen of the twenty essential amino acids analyzed as metabolites also differed significantly. All amino acids were involved in protein digestion, absorption, and metabolism. All 16 amino acid contents were higher in Japanese yellowtail than in Korean yellowtail, except for glutamine. The fasting period was found to be the biggest factor contributing to the difference in amino acid contents, in addition to environmental factors (including feeding habits). These significant differences indicated that metabolomics could be used to determine geographical origin.

## 1. Introduction

Yellowtail (*Seriola quinqueradiata*) is a species belonging to Carangidae in Perciformes; it is a migratory fish that is widely distributed from the East China Sea to Hokkaido in Japan, including the oceans of Korea [1,2]. It is considered a high-quality fish that is popular in both Korea and Japan; the demand for yellowtail has increased during the last 10 years. In Korea, increased demand for yellowtail in winter leads to fish being imported from Japan. As the demand for yellowtail has increased over the last seven years, the import volume has increased from 162 tons to nearly 2560 tons [3]. However, consumers in Korea are increasingly wary of radiation-contaminated seafood from Japan because of the Fukushima Daiichi nuclear disaster. Despite consumers’ concern, there was an incident involving the deceptive sale of Japanese yellowtail as Korean yellowtail in 2020. To resolve consumers’ concern regarding the origin of seafood, a scientific method for determining its origin is needed.

There are many technologies for identifying the origins of food. Many physiochemical materials can be analyzed by different devices. For example, differences in isotopes can be measured by nuclear magnetic resonance (NMR) and infrared spectroscopy (IR), minerals and heavy metals can be investigated by atomic absorption spectroscopy (AAS), specific materials can be measured by high-performance liquid chromatography (HPLC), and trace elements and volatile compounds can be measured by gas chromatography (GC) [4]. Furthermore, electronic nose technology can be used, and deoxyribonucleic acid (DNA) can be measured through polymerase chain reaction (PCR). These techniques have all been used to identify the origins of food. However, it is difficult to analyze and compare genetic information between fish of the same species. Agricultural products have specific characteristics derived from the distinct climatic characteristics and soil compositions of their places of origin. For example, the origins of rice, wine, and coffee have been distinguished by analyzing trace minerals using inductively coupled plasma–mass spectrometry (ICP/MS) [4]. Jung et al. discriminated the origins of beef from Australia, New Zealand, the USA, and Korea through ^1^H NMR analysis [5]. The origin of lamb has also been determined by analyzing the accumulation of elements in animal tissues. However, this process is relatively complicated [6].

Many methods can distinguish the geographical origins of seafood. However, most of these analysis methods are based on DNA, and methods for distinguishing the origins of seafood have exhibited no significant differences between samples from different countries. Random amplified polymorphic DNA (RAPD) PCR technology has been used to discriminate between flatfish and yellow corbina [7,8]. Some species have been discriminated from each other using technology based on DNA, but this DNA method has also failed for other species. In the present study, metabolomics was used to develop biomarkers for identifying the origin of yellowtail. Metabolomics constitutes investigating all metabolites from a living organism. Metabolites are the final products of cellular regulatory processes. Metabolomics is a new omics technology in the area of discriminating seafood origin that can analyze cellular functions throughout entire metabolic pathways [9,10]. Metabolomics allows for the thorough investigation of the biochemical activities related to genetics and environmental factors. Recently, metabolomics has been used in various fields, such as medicine and diet, as well as the relationship between disease and health. For example, metabolomic analysis has been used to identify extra-virgin olive oils and sesame seeds from different geographical origins [11,12]. It has also been used to identify the origins of sea cucumbers using liquid chromatography/mass spectroscopy (LC/MS) [13]. Though many kinds of metabolomic analyses have been conducted to determine the geographical origins of seafood, to date, no such studies have focused on fish.

The metabolites and physiochemical products of organisms vary depending on the environments in which they grow. As aquatic organisms generally experience less severe environmental variations than terrestrial organisms, determining their geographical origins is relatively difficult. Therefore, this study aimed to discriminate the country of origin of yellowtail by analyzing their tissues via metabolomics technology using capillary electrophoresis–time of flight/mass spectrometry (CE-TOF/MS). These metabolic differences were investigated through metabolomic analysis to evaluate their potential as biomarkers for determining geographical origin.

## 2. Results and Discussion

### 2.1. Metabolomics Profile

CE-TOF/MS analysis of the metabolite profiles of Korean (*S. quinqueradiata* Korean, SQK; *n* = 10) and Japanese (*S. quinqueradiata* Japanese, SQJ; *n* = 10) yellowtail in two different modes (cationic and anionic) detected 233 peaks (171 in the cation mode and 62 in the anion mode). Putative metabolites were annotated based on the Human Metabolome Technologies Inc. (HMT) standard library and the known–unknown peak library, referring to mass-to-charge ratio (*m*/*z*) and migration time (MT). There were no unknown peaks; all peaks could be matched to these libraries.

**Figure 1:**
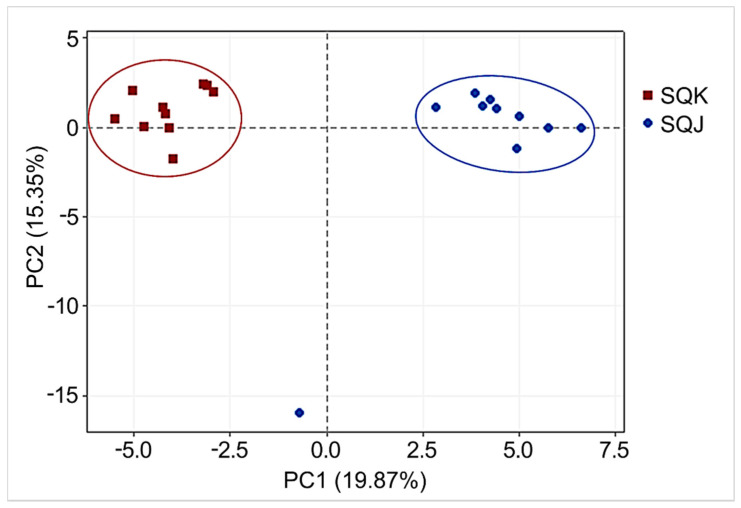
Principal component analysis (PCA) results. PC1 and PC2 show the first and second principal components, respectively. The numbers in parentheses show the contribution rates. The red dots indicate Korean yellowtail (*S. quinqueradiata* Korean, SQK; *n* = 10) and the blue dots indicate Japanese yellowtail (*S. quinqueradiata* Japanese, SQJ; *n* = 10).

**Figure 2:**
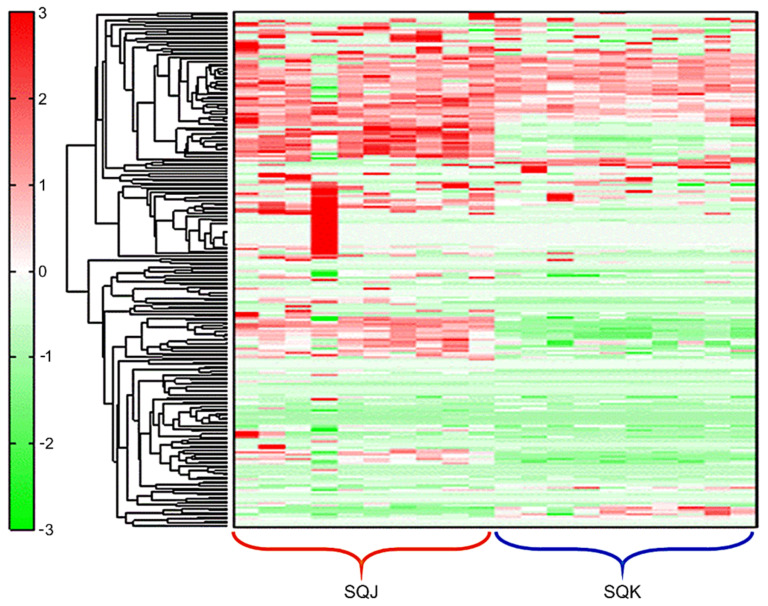
A heat map of hierarchical cluster analysis (HCA) comparing metabolites between SQJ and SQK. The y-axis shows peaks determined by HCA. The distances between peaks are displayed in a tree diagram; the degrees of red and green color indicate higher and lower contents of metabolites than the average, respectively.

Multivariate statistical analysis identified two completely separate groups (SQK and SQJ). The results of principal component analysis (PCA) of the two groups are depicted in Figure 1. These two groups are determined in this graph, except for one sample of SQJ. Hierarchical cluster analysis (HCA) based on putative metabolites is shown in Figure 2.

The analysis highlighted two notable differences, the first being related to hypoxanthine, guanosine, uric acid, fumaric acid, pyruvic acid, and lysine, and the second being related to essential amino acids such as tyrosine, tryptophan, methionine, phenylalanine, leucine, isoleucine, and organic acids (such as malic and citric acids).

In a prior study of the geographical origin of tobacco, PCA conducted on metabolic analysis using gas chromatography/mass spectrometry (GC/MS) and capillary electrophoresis–mass spectrometry (CE/MS) showed evident discrimination [14]. The geographical origins of sea cucumbers from four different regions have also been identified by ultra-high performance liquid chromatography–quadrupole-time of flight/mass spectroscopy (UPLC-Q-TOF/MS) metabolomics [13]. Although there were differences between these studies and the present study, such as the species and analysis devices, they all appear to show that metabolomics can determine the geographical origins of living organisms. Of all of the metabolites that were detected here via CE-TOF/MS analysis, 80 metabolites were quantified (49 cationic and 31 anionic). It would be judged that the PCA model could provide a clear standard for discriminating between Korean and Japanese yellowtail by increasing the number of samples. In addition, this analysis data would be proven and verified through additional analysis by season and fish size.

### 2.2. Differences in Organic Acid Metabolites in the Tricarboxylic Acid (TCA) Cycle

**Figure 3:**
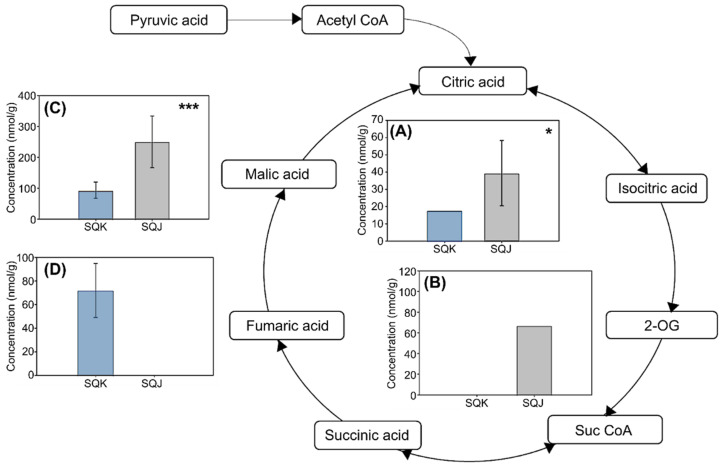
Different metabolite compounds in the TCA cycle: (**A**) citric acid. (**B**) 2-oxaloglutaric acid, (**C**) fumaric acid, and (**D**) malic acid. For each plot, blue bar = SQK and gray bar = SQJ. Error bars represent standard deviation. Significant differences were calculated using Welch’s test (* < 0.05, *** < 0.001).

The TCA cycle is the primary process that produces energy resources in the form of adenosine triphosphate (ATP). Pyruvic acid turns into acetyl coenzyme A (acetyl-CoA) through a decarbonation reaction. The TCA cycle starts with the conversion of citric acid to oxaloacetic acid; it produces 15 ATP [15]. There are many organic acids in the TCA cycle, such as isocitric, citric, succinic, fumaric, malic, and oxaloacetic acids. In the present study, citric, fumaric, and malic acids showed significant differences in their concentrations following metabolite analysis, as shown in Figure 3.

The first step of the TCA cycle comprises an aldol condensation between acetyl CoA and oxaloacetate; this produces a thioester intermediate. Citric acid and coenzyme A are then produced via the hydrolysis of this thioester. In the TCA cycle, citric acid then turns into an isomer, isocitric acid [16]. Here, citric acid showed a higher content in SQJ (39 ± 19 nmol·g^−1^) than in SQK (19 ± 0 nmol·g^−1^). Fumaric acid is one of the isomers of unsaturated dicarboxylic acid; it is an intermediate in the TCA cycle, which cells use to generate energy in the form of ATP. As fumaric acid is also a component in the urea cycle, it is a connecting component between the TCA and urea cycles [17]. Here, fumaric acid was not detected in SQJ, but was present at a concentration of 72 ± 23 nmol·g^−1^ in SQK. Malic acid is also an intermediate in the TCA cycle; it is produced by the addition of a hydroxyl group to fumaric acid, and from pyruvic acid via an anaplerotic reaction. Here, the malic acid concentration was higher in SQK than in SQJ. However, succinic acid was not detected in either SQJ or SQK.

SQJ had higher contents than SQK for all acids except for fumaric acid. Substances with different contents in the TCA cycle were judged to bring about the observed differences in geographical location and physical distance from the place of origin. Live fish do not feed during transportation because the fish with full digestive tracts produce excretion, which makes the water dirty and need more oxygen [18]. In addition, the yellowtail fish were not fed during transportation, which could have affected the metabolites in their tissue cells. SQJ underwent a longer fasting period than SQK. If they are not fed, living organisms try to produce energy using stored compounds. Amino acids are transformed into glucose through organic acids [19]. For the yellowtail featured here, especially SQJ, it was judged that stored nutrition material and components were decomposed into organic acids and glucose to be usable for glycolysis, as well as in the TCA cycle (for energy production). This pathway starts with amino acids, which are transformed into glucose via organic acids.

Additionally, differences in feeding materials and methods could be the reason for the observed differences. Both countries cultivate yellowtail that were caught in the East China Sea or the southern sea area of the Japanese archipelago as juveniles. The juvenile catching spots are geographically close to each other, but the aquaculture methods used by each country are different. Sardine, horse mackerel, dried fish meal, soybean meal, and frozen fish are all used as foodstuffs during the aquaculture of yellowtail [20,21]. Both countries featured here use similar feeding practices, but there may have been differences in feeding materials and patterns for yellowtail [22]. Frozen minced sardine was provided as feeding material to yellowtail in both Japan and Korea. Japanese yellowtail consumed extruded pellets with higher carbohydrate and lipid content than Korean yellowtail before harvesting. The carbohydrate and lipid may have been stored in the form of glycogen and converted into glucose for energy production [23]. Flounder fed extruded pellets had more glycine, glutamic acid, alanine, and arginine than flounder fed moist pellets [24]. Similarly, Japanese yellowtail that consumed extruded pellets contained higher amounts of amino acid. 

### 2.3. Differences in Metabolites in Amino Acid Metabolism

Amino acids are mainly observed in two metabolic pathways: the metabolism of each amino acid and protein digestion and absorption. They have also been shown to be involved in many other metabolisms, such as the ATP binding cassette (ABC) transporter pathway and nucleic acid metabolism. Many fish have relatively high protein contents and essential amino acid requirements [25]. Proteins and amino acids are both essential components in foodstuffs for fish. Trout have been shown to consume 70% of their dietary calories from protein [26]. Protein is decomposed into amino acids through digestion, and amino acids are either stored or consumed for energy production. In the absence of feeding, stored amino acids will be converted into glucose through organic acids. Research into fasting cattle showed that their oxygen consumption decreased during fasting, and that 23–26% of their total energy loss was derived from protein oxidation [27]. Amino acids are produced through protein oxidation as an energy source in glycolysis and in the TCA cycle.

**Figure 4:**
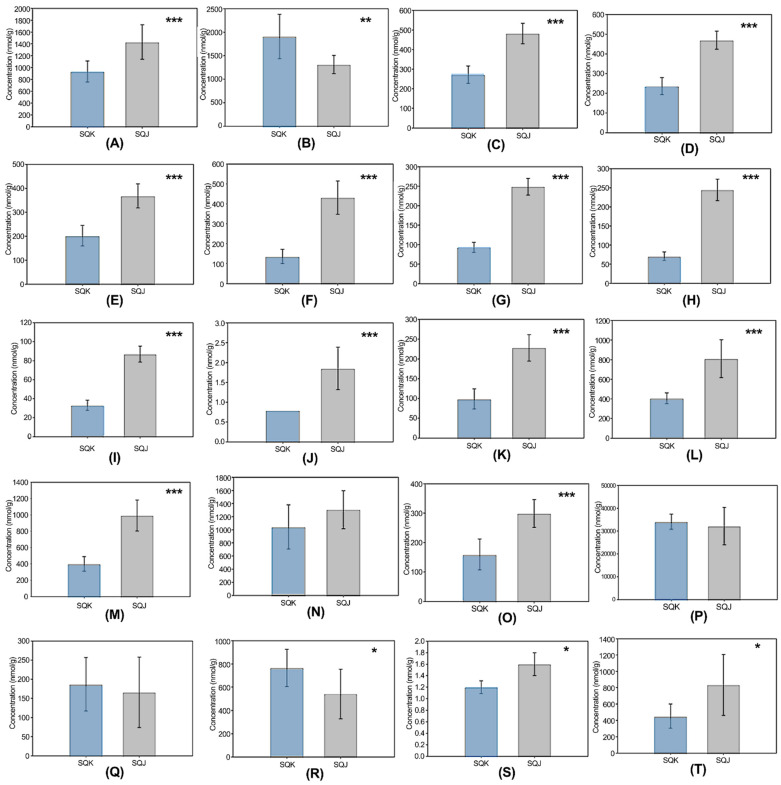
Differences observed in metabolite analysis of 20 amino acids between SQK (*n* = 10, blue bars) and SQJ (*n* = 10, gray bars): (**A**) glycine, (**B**) alanine, (**C**) valine, (**D**) leucine, (**E**) isoleucine, (**F**) proline, (**G**) phenylalanine, (**H**) tyrosine, (**I**) tryptophan, (**J**) cysteine, (**K**) methionine, (**L**) serine, (**M**) threonine, (**N**) lysine, (**O**) arginine, (**P**) histidine, (**Q**) aspartate, (**R**) glutamate, (**S**) asparagine, and (**T**) glutamine. Error bars represent standard deviation. Significant differences were calculated using Welch’s test (* < 0.05, ** < 0.01, and *** < 0.001).

The differences in metabolites in protein and amino acid metabolism are described in Figure 4. Twenty amino acids were detected and quantified; all but four (cysteine, lysine, histidine, and aspartate) showed significant differences between fish. Most of the investigated amino acids were more concentrated in SQJ than in SQK, except for alanine, histidine, aspartate, and glutamate, which were not.

A study that compared the nutritional components of feeding and starving red sea bream showed similar findings to those obtained here. In this study, most of the free amino acids showed higher concentrations in starving fish than in fed fish, although the present approach differed, as only the nutritional components of edible parts were examined [28]. Similarly, here, it was judged that different feeding practices considerably affected the observed differences in amino acid concentrations, as SQJ, which experienced a longer time without feeding during transportation, exhibited higher amino acid levels in metabolomic analysis. Additionally, quantified amino acids such as 2-aminobutyric acid, ethanolamine, and cystathionine exhibited significant differences, with their concentrations and relative contents being higher in SQJ than in SQK. Similarly, free amino acids (aminobutyric acid, ethanolamine, and cystathionine) in the edible parts of starving fish have been shown to be more concentrated than in fed fish [28]. It could be concluded that fewer amino acids enter the muscle tissues of non-starved fish than enter into other organs. Stored protein turned into amino acid, which the fish used as an energy source due to the lack of carbohydrate and lipid. This may be why Japanese yellowtail had higher amino acid contents. The lipogenesis of gluconeogenesis from amino acids does not play a significant role in the development of fish muscle tissue [29,30,31,32]. Here, the feeding state, feeding material, and environmental situation were the reasons for the different amino acid contents. In Korea, sardine and mackerel are the main feed for yellowtail in aquaculture, whereas in Japan, artificial feed is also used (including granulated feed, soybean meal moist pellet, and high fat dry pellets) because of a recent decrease in sardine yields [21,22]. In Japan, adult yellowtail were fed extruded pellets containing 20% lipids and 20% protein. In addition, the differences in water temperature between the two countries likely affected the amount and type of food species that were naturally available [2]. All of the above factors likely affected the observed metabolite differences.

### 2.4. Differences in Metabolites in Other Quantified Compounds

Other characteristic metabolites among the quantified compounds also exhibited significant differences. Table 1 shows the different metabolite contents of SQJ and SQK. Guanosine, hypoxanthine, and guanine are involved in purine metabolism. Sarcosine is related to glycine, serine, and threonine metabolisms. The contents of these four compounds were higher in SQJ than in SQK. As sarcosine is related to amino acid metabolism, its difference was likely derived from the observed amino acid differences discussed above. Although the relationship between purine metabolism and geographical difference has not yet been elucidated, it is possible that purine metabolism could be used as a biomarker following the confirmation of this relationship.

## 3. Materials and Methods

### 3.1. Sample Collection and Preparation

**Figure 5:**
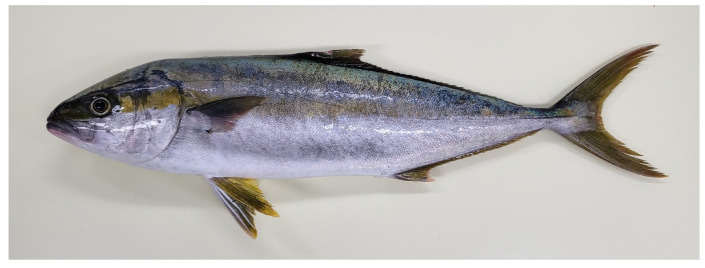
Yellowtail (*Seriola quinqueradiata*).

Ten SQK and ten SQJ were purchased from the supplier in October 2020. Each sample’s full length and weight were measured by the food supplier (Asia fish market, Busan, Korea). These full lengths and weights are shown in Table 2. The yellowtail was shown in Figure 5. All yellowtail samples were cultivated in either Korea or Japan. The Korean yellowtail were grown around Tong-young, Gyeongsangnam-do, and the Japanese yellowtail were cultivated in the Kyushu area [33].

Muscle tissue samples were taken. Muscle tissue was taken from the middle part between the lateral line and the dorsal fin, with the pectoral fin being used as criteria. The collected samples were immediately frozen using liquid nitrogen and stored at −80 °C.

### 3.2. Metabolite Extraction

Approximately 30 mg of muscle tissue sample was mixed with 750 µL of 50% acetonitrile in water (*v*/*v*) containing methionine sulfone and camphor-10-sulfonic acid as an internal standard (20 μM) and was homogenized at 3500 rpm (60 s × 5). The same quantity of 50% acetonitrile in water (*v*/*v*) was then added to the homogenized sample. Subsequently, 400 µL of supernatant was filtered using a 5-kDa cut-off filter (Ultrafree-MC-PLHCC; Human Metabolome Technologies, Yamagata, Japan) to remove macromolecules. The filtrate was concentrated via centrifugation and resuspended in 50 µL of ultrapure water immediately before analysis.

### 3.3. CE-TOF/MS Analysis

The extracted metabolites were measured in the cation and anion modes of CE-TOF/MS (Agilent CE-TOF/MS system, Agilent Technologies Inc., Santa Clara, CA, USA) based on the metabolome analysis conditions presented in Table 3 [34]. The samples were diluted ten times with ultrapure water for anion analysis; no dilution was conducted for cation analysis.

### 3.4. Metabolomics Profile Analysis

Peaks detected by CE-TOF/MS were processed using automatic integration software (MasterHands v2.18.0.1, developed at Keio University, Tokyo, Japan) to obtain peak information, including m/z, migration time (MT), and peak area. Putative metabolites were detected using the HMT’s standard library. The peak area was converted to relative peak area based on the metabolite peak area, internal standard peak area, and sample amount. All of the metabolite concentrations were calculated by normalizing the peak area of each metabolite with respect to the area of the internal standard, and by using standard curves, which were obtained by single point (100 or 50 μM) calibration.

### 3.5. Statistics Analysis

HCA was performed using statistical analysis software (Minitab 19.0, Minitab Inc., State College, PA, USA), and PCA was performed by SampleStat ver. 3.14 (HMT, Tsuruoka, Japan).

## 4. Conclusions

Misleading labeling practices regarding origin information can be used to deceive consumers. These practices are conducted because of the health concerns of consumers and economic aspects. Here, yellowtail fish from two different origins were investigated via metabolomic analysis, which revealed differences between Korean and Japanese yellowtail in the levels of some metabolites, such as malic acid, leucine, proline, tyrosine, serine, and cystathionine. These differences appear to derive from the differing fasting periods during transportation experienced by the two groups of fish, as well as from differences in their environmental states. In addition, different aquaculture methods, such as feeding material and juvenile catching time, affected the content of analyzed metabolites. The higher amino acid content of Japanese yellowtail was due to extruded feeding pellets. Statistical analysis revealed that the country of origin (Korea or Japan) could be determined. This validates that these biomarkers can be used to determine the geographical origins of fish. Differences in aquaculture methods and transportation periods derived from geographical distance have been identified as the primary reasons for differences between the metabolomics profiles of Japanese and Korean yellowtail fish. Metabolomics could therefore become a powerful tool for discriminating the geographical origins of various foodstuffs, as well as aquatic products.

## Figures and Tables

**Table 1:** Concentrations of other metabolites.

	Concentration (nmol·g^−1^)		Comparative Analysis	
	SQK	SQJ	Ratio ^2^	*p*-Value ^3^
Guanosine	10 ± 2 ^1^	22 ± 6.7	2.2	0.00023 ***
Hypoxanthine	55 ± 22	120 ± 79	2.2	0.031 *
Guanine	1.0 ± 0.2	2.9 ± 1.6	2.8	0.006 **
Sarcosine	3.5 ± 1.2	13 ± 4.0	3.6	0.000028 ***

^1^ Mean ± standard deviation. ^2^ Ratios were calculated using average detection values; SQK was used as the denominator. ^3^ *p*-values were calculated using Welch’s *t*-test (* < 0.05, ** < 0.01, and *** < 0.001).

**Table 2:** The sample information.

	SQK		SQJ	
Sample Number	Full Length (cm)	Weight (kg)	Full Length (cm)	Weight (kg)
1	84.0	4.92	74.0	4.46
2	66.5	3.95	78.2	4.94
3	71.1	3.99	75.0	4.85
4	72.3	4.15	75.6	4.10
5	71.9	4.12	79.0	5.50
6	70.9	3.93	80.8	5.40
7	71.8	4.14	77.0	4.97
8	68.0	3.86	76.4	4.58
9	72.6	4.06	77.0	3.98
10	72.7	4.56	73.8	4.07
Mean ± S.D.	72.7 ± 4.63	4.2 ± 0.33	76.7 ± 2.22	4.7 ± 0.54

**Table 3:** Conditions of CE-TOF/MS analysis (cation and anion modes).

	Cationic Metabolites (Cation Mode)	Anionic Metabolites (Anion Mode)
Capillary	fused silica capillary of 50 μm × 80 cm	
Run buffer	Cation Buffer Soln. (p/n: H3301-1001)	Anion Buffer Soln. (p/n: I3302-1023)
Rinse buffer	Cation Buffer Soln. (p/n: H3301-1001)	Anion buffer Soln. (p/n: I3302-1023)
Sample injection	Pressure injection 55 mbar, 10 s	Pressure injection 50 mbar, 22 s
CE voltage	Positive, 30 kV	Positive, 30 kV
MS ionization	Electrospray ionization (ESI) Positive	ESI negative
MS Capillary voltage	4000 V	3500 V
MS scan range	*m/z* 50–1000	*m/z* 50–1000
Sheath liquid	HMT Sheath Liquid (p/n: H3301-1021)	HMT Sheath Liquid (p/n: H3301-1021)

## Data Availability

The data presented in this study are available in the article.
